# *Duguetia pycnastera* Sandwith (Annonaceae) Leaf Essential Oil Inhibits HepG2 Cell Growth In Vitro and In Vivo

**DOI:** 10.3390/molecules27175664

**Published:** 2022-09-02

**Authors:** Emmanoel V. Costa, César A. S. de Souza, Alexandre F. C. Galvão, Valdenizia R. Silva, Luciano de S. Santos, Rosane B. Dias, Clarissa A. Gurgel Rocha, Milena B. P. Soares, Felipe M. A. da Silva, Hector H. F. Koolen, Daniel P. Bezerra

**Affiliations:** 1 Department of Chemistry, Federal University of Amazonas (UFAM), Manaus 69080-900, AM, Brazil; 2 Gonçalo Moniz Institute, Oswaldo Cruz Foundation (IGM-FIOCRUZ/BA), Salvador 40296-710, BA, Brazil; 3 Department of Propedeutics, School of Dentistry of the Federal University of Bahia, Salvador 40110-909, BA, Brazil; 4 SENAI Institute of Innovation (ISI) in Health Advanced Systems, University Center SENAI/CIMATEC, Salvador 41650-010, BA, Brazil; 5 Metabolomics and Mass Spectrometry Research Group, Amazonas State University (UEA), Manaus 690065-130, AM, Brazil

**Keywords:** *Duguetia pycnastera*, essential oil, HepG2 cells, cytotoxic, antitumor

## Abstract

*Duguetia pycnastera* Sandwith (Annonaceae) is a tropical tree that can be found in the Guyanas, Bolivia, Venezuela, and Brazil. In Brazil, it is popularly known as “ata”, “envira”, “envira-preta”, and “envira-surucucu”. In the present work, we investigated the in vitro and in vivo HepG2 cell growth inhibition capacity of *D. pycnastera* leaf essential oil (EO). The chemical composition of the EO was determined by GC–MS and GC–FID analyses. The alamar blue assay was used to examine the in vitro cytotoxicity of EO in cancer cell lines and non-cancerous cells. In EO-treated HepG2 cells, DNA fragmentation was measured by flow cytometry. The in vivo antitumor activity of the EO was assessed in C.B-17 SCID mice xenografted with HepG2 cells treated with the EO at a dosage of 40 mg/kg. Chemical composition analysis displayed the sesquiterpenes α-gurjunene (26.83%), bicyclogermacrene (24.90%), germacrene D (15.35%), and spathulenol (12.97%) as the main EO constituents. The EO exhibited cytotoxicity, with IC_50_ values ranging from 3.28 to 39.39 μg/mL in the cancer cell lines SCC4 and CAL27, respectively. The cytotoxic activity of the EO in non-cancerous cells revealed IC_50_ values of 16.57, 21.28, and >50 μg/mL for MRC-5, PBMC, and BJ cells, respectively. An increase of the fragmented DNA content was observed in EO-treated HepG2 cells. In vivo, EO displayed tumor mass inhibition activity by 47.76%. These findings imply that *D. pycnastera* leaf EO may have anti-liver cancer properties.

## 1. Introduction

Cancer is a major public health issue, with 19.3 million new cancer cases and approximately 10 million cancer deaths worldwide in 2020 [1]. The high incidence of cases suggests that research into early cancer detection and new cancer therapies are still urgently needed.

Plants in the genus *Duguetia* (Annonaceae) are known as sources of cytotoxic and antitumor substances and include promising species such as *Duguetia hadrantha* (Diels) R. E. Fr. [2], *Duguetia odorata* (Diels) J. F. Macbr. [3], *Duguetia glabriuscula* (R. E. Fr.) R. E. Fr. [4], *Duguetia furfuracea* (A. St.-Hil.) Saff. [5], *Duguetia gardneriana* Mart. [6], *Duguetia surinamensis* R. E. Fr. [7], and *Duguetia pycnastera* Sandwith [8].

*D. pycnastera* is a tropical tree, 3–10 m tall and 5–25 cm in diameter, native to Guyana, Bolivia, Venezuela, and Brazil. In Brazil, this species is commonly found in the Amazonas, Amapá, and Pará states of the Amazon rainforest. Popularly, it is known as “ata”, “envira”, “envira-preta”, and “envira-surucucu” [9]. *D. pycnastera* is distinguished by its bullate leaves. The flowering season occurs in April–October, and the fruiting season is from May to January. The fruits are considered edible [9], although they are still little known.

In Guyana folk medicine, the inner bark of *D. pycnastera* is scraped, macerated in water for 24 h, and drunk as a remedy to treat colds. The warmed bark can also be used as a poultice to treat muscle aches and pains, as well as coughs and colds. The outer bark decoction may also be used to treat coughs symptoms. The leaves can also be macerated in water to provide a folk medicine used to treat fevers or an herbal bath for body washing as a chills treatment [10].

Previous chemical investigation of the bark extract of *D. pycnastera* led to the isolation of benzenoids, such as 2,4,5-trimethoxy-styrene and γ-asarone, and isoquinoline alkaloids, such as nornuciferidine, lysicamine, guatterine *N*-oxide, *O*-methylmoschatoline, and (*S*)-reticuline. Among the latter, lysicamine showed cytotoxicity against melanoma, leukemia, and liver cancer cells [8]. On the other hand, when studying the leaves, the alkaloids *O*-methylisopiline, anonaine, isopiline, nornuciferine, norstephalagine, liriodenine, *O*-methylmoschatoline, lysicamine, and isocorypalmine and the terpene loliolide were isolated as the main compounds [11]. In a previous study, the essential oil (EO) from the leaves and stems of this species displayed *allo*-aromadendrene, spathulenol, elemol and germacrene D as its main constituents [12]. However, the authors did not describe the chemical composition of the EOs obtained from each separate part (leaves and stems), as they focused on the EO derived from the combination of stems and leaves. In this study, the ability of *D. pycnastera* leaf EO to inhibit HepG2 cell growth in vitro and in vivo was investigated.

## 2. Results and Discussion

### 2.1. Chemical Composition of D. pycnastera Leaf EO

**Figure 1:**
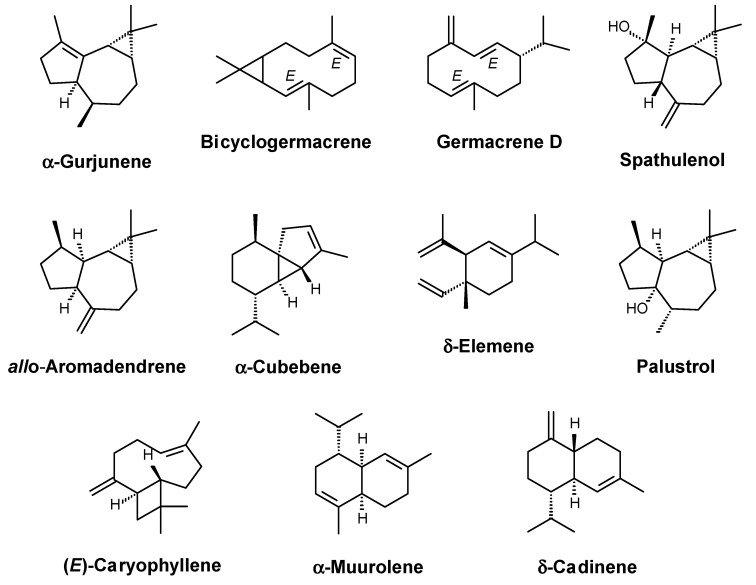
Main compounds identified in *D. pycnastera* leaf EO.

The chemical composition of *D. pycnastera* leaf EO was determined using GC–MS and GC–FID analyses. The EO samples presented a yellowish coloration, with a yield of 0.12 ± 0.02% in relation to the weight of the dried material. The chemical compounds were identified using their mass spectra (Figures S1–S12), arithmetic index (AI), and a comparison with published data. A total of 23 compounds were annotated, accounting for 96.75% of the EO composition (Table 1). Only terpenoids were identified among the detected compounds, with sesquiterpenes dominating (hydrocarbons and oxygenated derivatives). Sesquiterpene hydrocarbons (20 substances) formed the dominant class, comprising 81.44% of the total composition. Three oxygenated sesquiterpenes corresponding to 15.31% of the total composition were identified, but only one of them (spathulenol) was in a significant amount (12.97%) (Table 1). Among the main substances, α-gurjunene (26.83%), bicyclogermacrene (24.90%), germacrene D (15.35%), and spathulenol (12.97%) dominated the EO of *D. pycnastera* (Figure 1; Table 1). Other compounds were identified, but in amounts below 2.5%: *allo*-aromadendrene (2.21%), α-cubebene (1.80%), δ-elemene (1.54%), palustrol (1.40%), (*E*)-caryophyllene (1.27%), α-muurolene (1.26%), and δ-cadinene (1.20%) (Figure 1; Table 1).

The presence of the major compounds and of the minor compounds agrees with a previous description of the chemical constituents of *Duguetia* species, corroborating the previously published data of the genus *Duguetia* [15]. Among the major compounds identified, the most representative and most often found in EOs of *Duguetia* species are germacrene D, bicyclogermacrene, and spathulenol, which together can be considered chemophenetic markers of the EO of *Duguetia* species, particularly in the leaf EO [15]. An interesting observation was the presence of α-gurjunene as the main constituent of the EO samples, which is also the principal compound in the stems of *D. furfuracea* [15]. Additionally, α-gurjunene has been described as a minor constituent in the EO of *Duguetia lanceolata* A. St.-Hil. [16] and *D. furfuracea* stem barks [17]. Furthermore, the report of this substance in the leaf EOs of *Duguetia* species is not common; so far, α-gurjunene has not been described. However, it is important to note that the chemical composition of EOs can change due to a variety of factors such as climate, geographical location, soil characteristics and fertilization level, and the year season.

### 2.2. D. pycnastera Leaf EO Has In Vitro Cytotoxic Activity

*D. pycnastera* leaf EO was found to be cytotoxic in vitro in 13 cancer cells (HepG2, NB4, JURKAT, THP-1, K562, HL-60, KG-1a, HCT116, MCF-7, SCC4, HSC-3, CAL27, and B16-F10) and 3 non-cancer cells (MRC-5, PBMC, and BJ). Table 2 displays the IC_50_ values that were discovered for the cancer cell lines, in which EO samples showed IC_50_ values ranging from 3.28 to 39.39 μg/mL, with the lower IC_50_ measured for SCC4 and the highest for CAL27. When we tested the cytotoxicity in non-cancerous cells, the EO revealed IC_50_ values of 16.57, 21.28, and more than 50 μg/mL for MRC-5, PBMC, and BJ cells, respectively. Doxorubicin showed IC_50_ values ranging from 0.01 to 1.45 μg/mL for the cancer cell lines SCC-4 and MCF-7 and IC_50_ values of 0.91, 3.04, and 0.55 μg/mL for the non-cancerous cells MRC-5, PBMC, and BJ, respectively.

EOs showing IC_50_ values lower than 30 μg/mL in cell viability assays using tumor cell lines are considered promising in our cytotoxic compound screening program [18,19,20]. Interestingly, *D. pycnastera* leaf EO had IC_50_ values lower than 30 μg/mL for many of the cell lines tested and was chosen for future experiments. This is the first evidence that this EO has cytotoxic activity.

In a previous study, the EO from *D. gardneriana* leaves was found to be cytotoxic to B16-F10, HepG2, HL-60, and K562 cell lines, with IC_50_ values of 16.9, 19.2, 13.1, and 19.3 μg/mL, respectively [6]. *D. gabriuscula* leaf EO was also found to be cytotoxic to human larynx carcinoma (Hep2) cells, with an IC_50_ value of 11.6 μg/mL [3]. Furthermore, the EOs of *D. lanceolata* bark and *D. furfuracea* stem were cytotoxic to *Artemia salina* [17,21]. Among the main components of *D. pycnastera* leaf EO, germacrene D, bicyclogermacrene, and spathulenol have been shown to be cytotoxic in different cancer cell lines [22,23,24].

**Figure 2:**
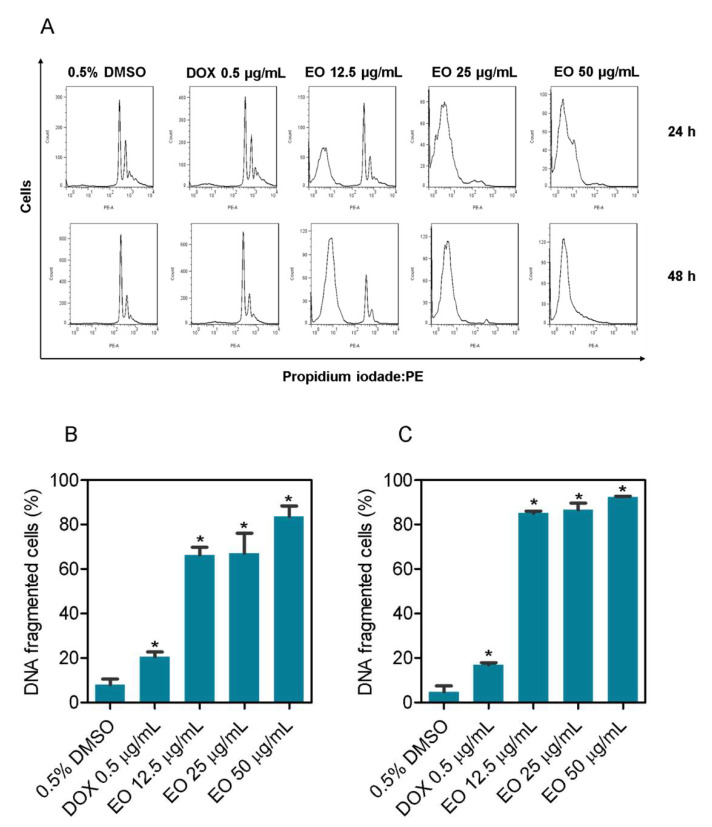
The effect of *D. pycnastera* leaf EO on HepG2 cell DNA fragmentation. (**A**) Representative cytometry histograms. (**B**) Internucleosomal DNA fragmentation after 24 h of treatment. (**C**) Internucleosomal DNA fragmentation after 48 h of treatment. The vehicle used to dilute the EO (0.5% DMSO) served as a negative control, and doxorubicin (DOX, 0.5 μg/mL) served as a positive control. The results are expressed as the average ± S.E.M. of three independent experiments performed in duplicate. * *p* < 0.05 when compared to the negative control using the two-tailed unpaired Student’s *t*-test.

To confirm the cytotoxic effects of *D. pycnastera* leaf EO, internucleosomal DNA fragmentation and cell cycle were measured in EO-treated HepG2 cells (Figure 2). All DNA that was sub-diploid (sub-G_1_) in size was considered fragmented. After 24 and 48 h of treatment with the EO, there was a significant increase in cells with fragmented DNA at all concentrations tested (12.5, 25, and 50 μg/mL). As a positive control, doxorubicin significantly increased the number of cells with fragmented DNA.

In the Annonaceae family, *Guatteria olivacea* R. E. Fr. leaf EO also caused DNA fragmentation and cell death by apoptosis in HepG2 cells. Interestingly, germacrene D, bicyclogermacrene, and spathulenol are among its main components [18]. The EO extracted from *Annona squamosa* L. pericarps presented spathulenol as its major chemical component and induced apoptosis in SMMC-7721 liver cancer cells [25]. *Annona vepretorum* Mart. leaf EO also has spathulenol as one of its major components and has been reported to cause apoptosis in B16-F10 cells [24]. Likewise, the EO of *Guatteria megalophylla* Diels leaves contains spathulenol among its main components and caused DNA fragmentation in the HL-60 cell line [26].

### 2.3. D. pycnastera Leaf EO Has an Antitumor Effect In Vivo

**Figure 3:**
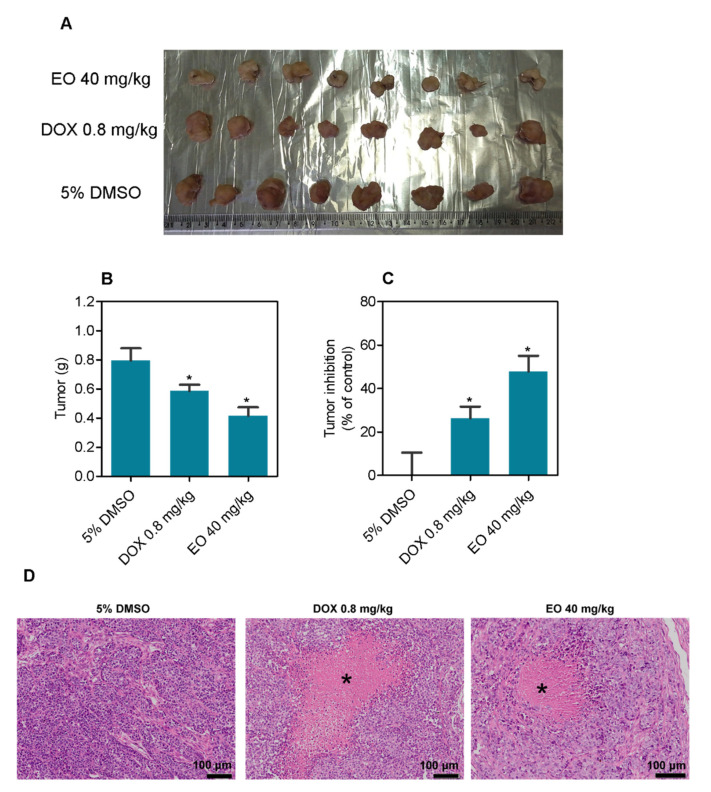
*D. pycnastera* leaf EO exerted an anti-liver cancer effect in HepG2 tumor-bearing mice. (**A**) Tumor images. (**B**) Post-treatment tumor weight (g). (**C**) Tumor inhibition (%) following treatment. (**D**) Representative histological analysis of the HepG2 tumors stained with hematoxylin and eosin and analyzed by light microscopy. The asterisks indicate areas of tissue necrosis. The vehicle used to dilute the EO (5% DMSO) served as a negative control, and doxorubicin (DOX, 0.8 mg/kg) served as a positive control. The results are expressed as the average ± S.E.M. of tumors from 8–9 animals. * *p* < 0.05 when compared to the negative control using the two-tailed unpaired Student’s *t*-test.

*D. pycnastera* leaf EO was tested for antitumor activity in CB-17 SCID mice transplanted with HepG2 cells. The mice were administered 40 mg/kg EO intraperitoneally once a day for 21 days (Figure 3). The mean weight of the tumors in negative control animals at the end of treatment was 0.80 ± 0.08 g. In EO-treated animals, the mean tumor weight was 0.42 ± 0.06 g. Tumor mass inhibition was 47.76%. Doxorubicin decreased tumor mass by 26.3%. HepG2 tumors were formed by highly proliferative and hyperchromatic epithelial-like cells. Pleomorphism and atypical mitosis were present in all experimental groups. While in EO-treated mice the tumor cells were arranged in compact nodules bounded by dense connective tissue, in negative control animals the cells were arranged in small patches of epithelial-like cells. Necrosis was more abundant in EO- and doxorubicin-treated animals.

**Figure 4:**
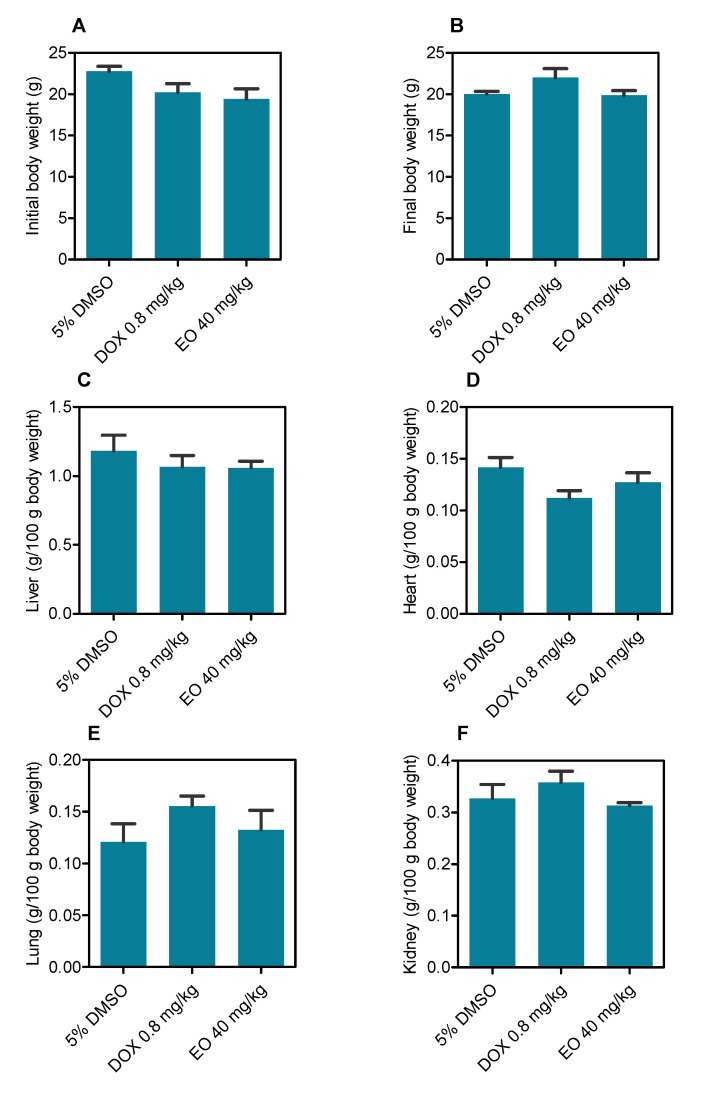
*D. pycnastera* leaf EO effects on the body and relative organ weight in HepG2 tumor-bearing mice. (**A**) Initial body weight (g). (**B**) Final body weight (g). (**C**) Liver (g/100 g of body weight). (**D**) Heart (g/100 g of body weight). (**E**) Lung (g/100 g of body weight). (**F**) Kidney (g/100 g of body weight). The vehicle used to dilute the EO (5% DMSO) served as a negative control, and doxorubicin (DOX, 0.8 mg/kg) served as a positive control. The results are expressed as the average ± S.E.M. of body and organ weights from 8–9 animals.

Regarding the toxicological aspects, no deaths were recorded during the treatment in any group, and no significant changes were observed in the body and organ weights in any group (*p* > 0.05) (Figure 4).

**Figure 5:**
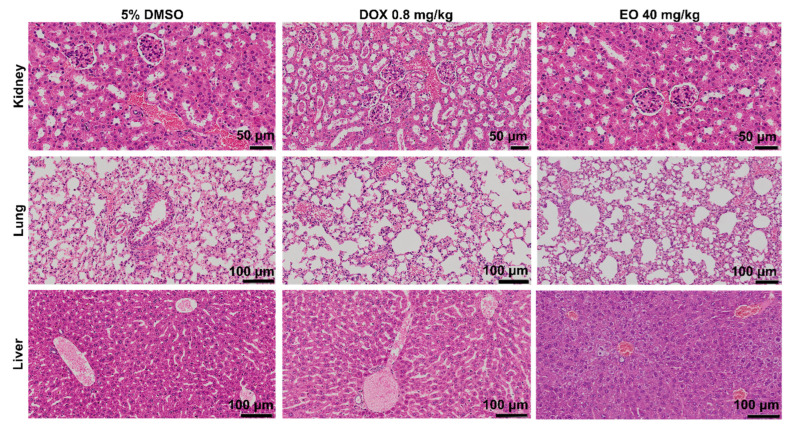
Representative photomicrographs of the kidney, lung, and liver of HepG2 tumor-bearing mice treated with *D. pycnastera* leaf EO. The vehicle used to dilute the EO (5% DMSO) served as a negative control, and doxorubicin (DOX) served as a positive control.

Histological analyses of the kidneys, livers, lungs, and hearts of the animals were performed using light microscopy (Figure 5). The animals in the present study presented a preserved renal architecture. However, focal areas of coagulation necrosis were observed in the tubules of the renal cortex of the animals treated with DOX and EO. In addition, all experimental groups showed moderate to severe vascular hyperemia and a slight decrease in Bowman’s space due to glomerular hyalinization. In the lungs, the architecture of the parenchyma ranged from preserved to partially preserved. This change in the lung architecture was related to the thickening of the alveolar septa and, consequently, pulmonary atelectasis. Other histopathological alterations were also observed, such as vascular hyperemia, polymorphonuclear infiltrate, edema, hemorrhage, and focal areas of hemosiderin deposition. The morphological changes ranged from mild to severe and were more noticeable in the animals administered the EO. The portal architecture was preserved in the livers. However, vascular hyperemia, hydropic degeneration, inflammatory cell infiltrate around the portal space and sinusoids, and focal areas of coagulation necrosis were found in the liver parenchyma. In addition, all EO-treated animals showed moderate microgotic steatosis. The hearts of all animals in this study presented no significant histopathological changes.

Previously, the leaf EO of *D. gardneriana* was tested in vivo on B16-F10 tumor-bearing C57BL/6 mice and found to reduce tumor growth by 5.4 and 37.5% at doses of 40 and 80 mg/kg, respectively [6]. In the Annonaceae family, HepG2-bearing C.B-17 SCID mice treated with *G. olivacea* leaf EO at doses of 20 and 40 mg/kg showed tumor reduction rates of 32.8–57.9% [18]. *A. vepretorum* leaf EO reduced the growth of B16-F12 in vivo by 34.46% at the dose of 50 mg/kg, which was increased to 62.66% when the EO was microencapsulated in β-cyclodextrin [24]. In C.B-17 SCID mice inoculated with HL-60 cells, *G. megalophylla* leaf EO reduced tumor growth by 16.6 and 48.8% at doses of 50 and 100 mg/kg [26]. Bicyclogermacrene and germacrene D were also found among the main chemical constituents of the leaf EOs of *Xylopia frutescens* Aubl., *Xylopia laevigata* (Mart.) R. E. Fr., and *Guatteria pogonopus* Mart. In sarcoma 180-bearing mice, the EO from the first plant inhibited tumor growth by 31.0 and 37.5%, that from the second by 37.3 and 42.5%, and that from the third by 25.3 and 42.6%, all at doses of 50 and 100 mg/kg [27,28,29].

## 3. Materials and Methods

### 3.1. Botanical Material

*D. pycnastera* leaves were collected on 19 September 2018 at the Adolpho Ducke Reserve (coordinates: 2°55′37.4″ S and 59°58′36.0″ W), Manaus, AM, Brazil, and identified by Prof. Antonio Carlos Webber, a plant taxonomist of the Department of Biology at the Federal University of Amazonas (UFAM). The herbarium received a voucher specimen (#10812). This study was entered into the Brazilian SISGEN database as A70EDCD.

### 3.2. Chemical Evaluation

#### 3.2.1. Essential Oil Extraction

*D. pycnastera* leaves were oven-dried with air circulation at 40 °C for 24 h before being subjected to a 4 h hydrodistillation using a Clevenger-type apparatus (Amitel, São Paulo, SP, Brazil). Hydrodistillation was performed twice (2 × 300 g = 600 g). The EO samples were dried with anhydrous sodium sulfate (Na_2_SO_4_), and the % of their content was obtained using the weight of the dry material used in each hydrodistillation. The standard deviation of the duplicate was then computed. Prior to chemical and biological analyses, the EO was frozen (−4 °C). To prepare the stock solution for pharmacological assays, 10 mg of EO was dissolved in 1 mL of dimethyl sulfoxide (DMSO, Vetec Quimica Fina Ltda., Duque de Caxias, RJ, Brazil).

#### 3.2.2. GC–FID and GC–MS Analyses

A Shimadzu GC-17A GC system with a DB-5MS capillary column (30 m × 0.25 mm × 0.25 μm) was used for gas chromatography with flame ionization detection (GC-FID) analysis, and a Trace Ultra gas chromatograph system coupled to an ISQ single quadrupole mass spectrometer (Thermo Scientific, Waltham, MA, USA) was used for gas chromatography with mass spectrometry (GC–MS) detection analysis, both according to our previous work [18,30].

The EO constituents were identified by comparing the obtained mass spectra to those in the NIST library, as well as by comparing the arithmetic index (AI) to previously published data [14]. To calculate the AI, a homologous series of linear hydrocarbons (C_8_–C_20_) were injected under the same analysis conditions, and the Van den Dool and Kratz equation [13] was used.

### 3.3. Pharmacological Evaluation

#### 3.3.1. In Vitro

##### Cells

The American Type Culture Collection (ATCC, Manassas, VA, USA) provided the cell line panel comprising the human liver cancer HepG2 cell line, the human leukemia NB4, THP-1, JURKAT, K562, HL-60, and KG-1a cell lines, the human breast cancer MCF-7 cell line, the human colon cancer HCT116 cell line, the human tongue cancer SCC4, CAL27, and HSC-3 cell lines, the mouse melanoma B16-F10 cell line, the human pulmonary fibroblast MRC-5 cell line, and the human foreskin fibroblast BJ cell lines. All cells were cultured according to the ATCC animal cell culture guide. The cells were routinely maintained in Roswell Park Memorial Institute (RPMI) 1640 or Dulbecco’s Modified Eagle Medium/Nutrient Mixture F-12 (DMEM-F12) medium with 10% or 20% fetal bovine serum (FBS) and 50 µg/mL of gentamicin and stored in a humidified atmosphere at 37 °C with 5% CO_2_. All cell lines used were free of mycoplasma infection as tested by a mycoplasma staining kit (Sigma-Aldrich, St. Louis, MO, USA).

The standard Ficoll density protocol was applied to obtain peripheral blood mononuclear cells (PBMC) from healthy donors. The PBMC were cultured in RPMI 1640 or DMEM-F12 medium with 20% FBS and 50 μg/mL of gentamicin and stored at 37 °C with 5% CO_2_. To stimulate cell division in T lymphocytes, concanavalin A (10 μg/mL, Sigma-Aldrich) was added as a mitogen at the start of the culture. The experimental protocols were approved by the Oswaldo Cruz Foundation’s Research Ethics Committee, Salvador, BA, Brazil (#031019/2013).

##### Alamar Blue Assay

Cell viability was analyzed using the alamar blue assay, as previously described [31,32,33]. In 96-well plates, exponentially growing cells were seeded at a density of 7 × 10^3^ cells/well for adherent cells or 3 × 10^4^ cells/well for non-adherent cells. The EO was added to each well in eight serial concentrations ranging from 0.39 to 50 μg/mL prior to incubating for 72 h. Doxorubicin (Laboratory IMA S.A.I.C., Buenos Aires, Argentina) was used as a positive control. Each well received 20 μL of resazurin solution (0.312 mg/mL) at the conclusion of the treatment (Sigma-Aldrich Co.). A SpectraMax 190 Microplate Reader (Molecular Devices, Sunnyvale, CA, USA) was used to examine the absorbances of each well at 570 and 600 nm. The inhibitory concentration of 50% (IC_50_) with 95% confidence intervals (95% CI) derived from nonlinear regressions was calculated.

##### Internucleosomal DNA Fragmentation and Cell Cycle Distribution

Internucleosomal DNA fragmentation and cell cycle analysis were performed according to [34]. The cells were collected, washed with saline solution, and stained with a propidium iodide probe using a hypotonic fluorochrome solution containing 2 μg/mL of PI, 0.1% triton X-100, 0.1% sodium citrate, and 100 μg/mL of RNAse (all from Sigma-Aldrich), at room temperature (in the dark), and cell fluorescence was measured using flow cytometry. Per sample, at least 10,000 events were recorded. The BD LSRFortessa cytometer and BD FACSDiva Software (BD Biosciences) were used. The data were analyzed with FlowJo Software 10 (FlowJo Lcc; Ashland, OR, USA). Cellular debris was excluded from the analysis.

### 3.4. In Vivo

#### 3.4.1. Animals

Two month-old mature male and females C.B-17 SCID mice (20–25 g) were supplied by and housed in the Gonçalo Moniz Institute-FIOCRUZ animal facilities (Salvador, Bahia, Brazil), according to the experimental protocol that was approved by a local animal ethics committee (#01/2021). All mice were fed a standard pellet diet (with free access to food and water) and subjected to an artificially illuminated room (12 h dark/light cycle).

#### 3.4.2. Human Liver Cancer Xenograft Model

After an acclimatization period, HepG2 cells (10^7^ cells/500 μL/SQ/animal) were inoculated into the left front armpit of the mice on day 0, as previously described [35,36,37]. On day 1, the animals were treated by the intraperitoneal route (200 μL/animal) once a day for 21 days. Three groups of animals were analyzed: group 1 received the vehicle (5% DMSO solution) used for diluting the EO (*n* = 9); group 2 received doxorubicin (0.8 mg/kg, *n* = 8); and group 3 received the EO at a dose of 40 mg/kg (*n* = 9). On day 22, the animals were euthanized with an anesthetic overdose (thiopental, 100 mg/kg), and the tumors were excised and weighed. The inhibition ratio (percent) was calculated as follows: inhibition ratio (percent) = [(A − B)/A] × 100, where A is the negative control’s average tumor weight, and B is the treated group’s tumor weight.

#### 3.4.3. Systemic Toxicity Assessment

All animals were weighed at the start and end of the experiment to assess toxicological features. The animals were monitored for abnormalities throughout the experiment. The livers, kidneys, lungs, and hearts were removed, weighed, and examined for color change, signs of gross lesion formation, and/or hemorrhaging and fixed in 4% formaldehyde, dehydrated in a graded alcohol series, cleaned in xylene, and embedded in paraffin wax. Each tissue was cut into 5 μm-thick slices, stained with hematoxylin–eosin and/or Periodic Acid–Schiff stain (liver and kidney), and histologically examined by optical microscopy.

### 3.5. Statistical Analysis

The results are expressed as the average of three repetitions (performed in duplicate) ± S.E.M./S.D. or as IC_50_ values with 95% CI. For the statistical analysis, the two-tailed unpaired Student’s *t*-test was used (*p* < 0.05) by GraphPad Prism (Intuitive Software for Science; San Diego, CA, USA).

## 4. Conclusions

*D. pycnastera* leaf EO presents α-gurjunene, bicyclogermacrene, germacrene D and spathulenol as its main constituents and has anti-liver cancer activity in HepG2 cells and HepG2 tumor-bearing mice, which can be attributed to the action of a combination of major and minor chemical constituents.

## Figures and Tables

**Table 1:** Chemical composition of *D. pycnastera* leaf EO.

Compounds		AI ^a^	AI ^b^	Peak Area%
1	δ-Elemene	1334	1335	1.54 ± 0.03
2	α-Cubebene	1347	1348	1.80 ± 0.02
3	Ciclosativene	1364	1369	0.36 ± 0.01
4	α-Ylangene	1373	1373	0.91 ± 0.03
5	β-Bourbonene	1381	1387	0.14 ± 0.01
6	β-Cubebene	1387	1387	0.24 ± 0.01
7	β-Elemene	1389	1389	0.43 ± 0.00
8	α-Gurjunene	1406	1409	26.83 ± 0.10
9	(*E*)-Caryophyllene	1415	1417	1.27 ± 0.01
10	β-Copaene	1425	1430	0.72 ± 0.01
11	α-Guaiene	1436	1437	0.43 ± 0.03
12	α-Humulene	1450	1452	0.42 ± 0.02
13	*allo*-Aromadendrene	1457	1458	2.21 ± 0.02
14	γ-Gurjunene	1474	1475	0.45 ± 0.06
15	Germacrene D	1478	1480	15.35 ± 0.01
16	Bicyclogermacrene	1493	1500	24.90 ± 0.03
17	α-Muurolene	1498	1500	1.26 ± 0.01
18	Viridiflorene	1503	1496	0.54 ± 0.01
19	γ-Cadinene	1511	1513	0.44 ± 0.01
20	δ-Cadinene	1520	1522	1.20 ± 0.10
21	Palustrol	1562	1567	1.40 ± 0.03
22	Spathulenol	1572	1577	12.97 ± 0.02
23	Viridiflorol	1596	1592	0.94 ± 0.02
Sesquiterpene hydrocarbons				81.44
Oxygenated sesquiterpenes				15.31
Total not identified				3.25
Total identified				96.75

AI ^a^ (arithmetic index) calculated on a TR-5MS capillary column (30 m × 0.25 mm × 0.25 µm) using a homologous series of normal alkanes, according to [13]. AI ^b^ according to [14]. The results are expressed as average ± S.D.

**Table 2:** Cytotoxic effect of *D. pycnastera* leaf EO.

Cells	Histological Type	IC_50_ and 95% CI (in μg/mL)	
		DOX	EO
**Cancer cells**			
HepG2	human hepatocellular carcinoma	0.09 0.06–0.12	11.70 6.10–22.43
NB4	human acute promyelocytic leukemia	0.05 0.03–0.07	9.23 8.38–10.17
THP-1	human monocytic leukemia	0.08 0.05–0.12	13.05 10.73–15.87
JURKAT	human acute T cell leukemia	0.03 0.02–0.05	8.01 6.94–9.24
K562	human chronic myelogenous leukemia	0.70 0.36–1.36	14.59 12.58–16.91
HL-60	human acute promyelocytic leukemia	0.05 0.03–0.10	19.74 15.62–24.95
KG-1a	human myeloid leukemia	0.01 0.01–0.11	20.75 16.59–25.96
MCF-7	human breast adenocarcinoma	1.45 1.00–2.11	32.85 22.47–48.03
HCT116	human colon carcinoma	0.06 0.03–0.12	15.51 12.39–19.41
SCC4	human oral squamous cell carcinoma	0.01 0.002–0.04	3.28 3.00–3.59
CAL27	human oral squamous cell carcinoma	0.65 0.26–1.65	39.39 27.37–56.68
HSC-3	human oral squamous cell carcinoma	0.66 0.49–0.87	30.95 21.01–45.60
B16-F10	mouse melanoma	0.28 0.23–0.35	28.20 21.52–36.96
**Non-cancerous cells**			
MRC-5	human lung fibroblast	0.91 0.30–2.73	16.57 12.91–21.28
PBMC	human peripheral blood mononuclear cells	0.67 0.48–0.94	21.28 17.72–25.56
BJ	human foreskin fibroblast	0.55 0.22–1.37	>50

The positive control was doxorubicin (DOX).

## Data Availability

Not applicable.

## Supplementary Materials

The following supporting information can be downloaded at: https://www.mdpi.com/article/10.3390/molecules27175664/s1. Mass spectra data (Figures S1–S16).

## Sample Availability

Samples of the EO are not available from the authors.
