# Supplementary material for: Duguetia pycnastera Sandwith (Annonaceae) Leaf Essential Oil Inhibits HepG2 Cell Growth In Vitro and In Vivo
Source: Molecules. 2022 Sep 2;27(17):5664. doi: 10.3390/molecules27175664 (PMC9458038; doi:10.3390/molecules27175664)
Supplement: Supplementary file 1 [file molecules-27-05664-s001.zip › molecules-1875137-supplementary.pdf]

Supplementary Material

Duguetia pycnastera Sandwith (Annonaceae) leaf essential oil inhibits HepG2 cell growth in vitro and in vivo

Emmanoel V. Costa[1,*], César A. S. de Souza[1], Alexandre F. C. Galvão[2], Valdenizia R. Silva[2], Luciano de S. Santos[2], Rosane B. Dias[2,3], Clarissa A. Gurgel Rocha[2,3], Milena B. P. Soares[2,4], Felipe M. A. da Silva[1], Hector H. F. Koolen[5], Daniel P. Bezerra[2,*]

[1]Department of Chemistry, Federal University of Amazonas (UFAM), Manaus, Amazonas, 69080-900, Brazil.

[2]Gonçalo Moniz Institute, Oswaldo Cruz Foundation (IGM-FIOCRUZ/BA), Salvador, Bahia, 40296-710, Brazil.

[3]Department of Propedeutics, School of Dentistry of the Federal University of Bahia, Salvador, Bahia, 40110-909, Brazil.

[4]SENAI Institute of Innovation (ISI) in Health Advanced Systems, University Center SENAI/CIMATEC, Salvador, Bahia, 41650-010, Brazil.

[5]Metabolomics and Mass Spectrometry Research Group, Amazonas State University (UEA), Manaus, Amazonas, 690065-130, Brazil.

*Corresponding authors:

E. V. Costa (e-mail: evc@ufam.edu.br); D. P. Bezerra (e-mail: daniel.bezerra@fiocruz.br); Tel./Fax: +55-92-3305-1181 Ramal 2870 (E.V. Costa); Tel./Fax: +55-71-3176-2272 (D. P. Bezerra).

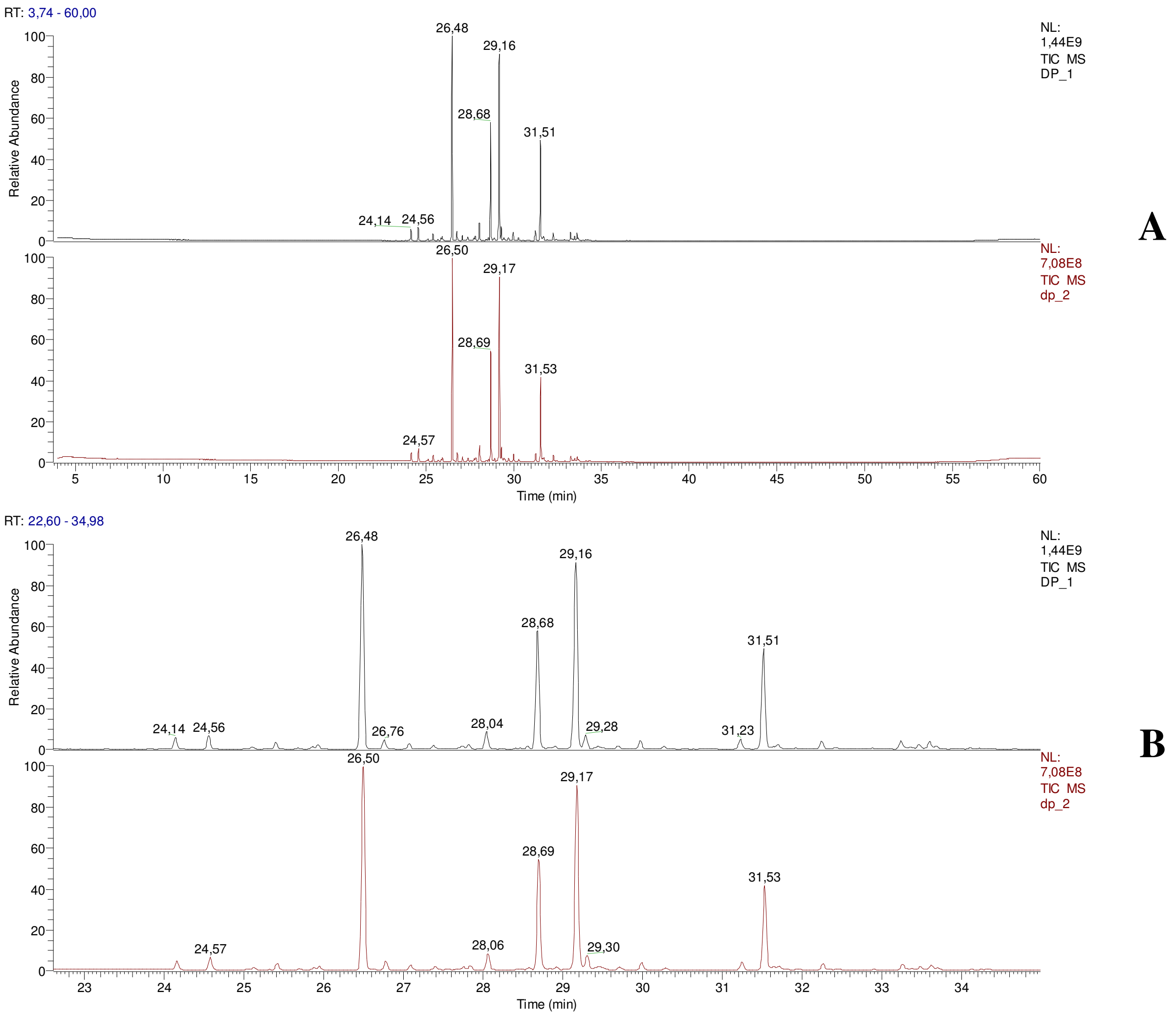

Figure S1. (A) Chromatogram of the total ions of the essential oil (triplicate) from the leaves of *Duguetia pycnastera*; (B) Enlargement of the region between 23.0 min to 34.0 min.

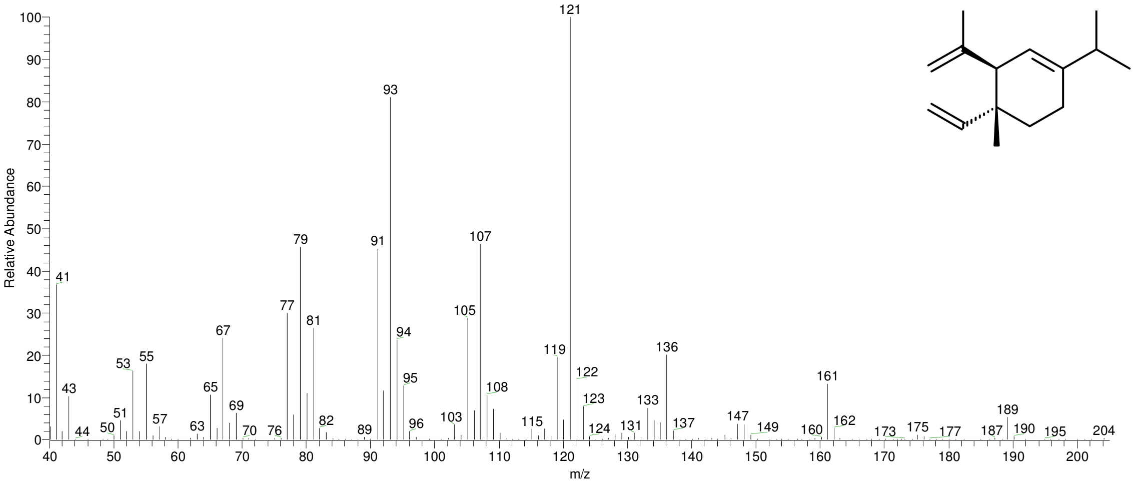

Figure S2. Mass spectrum of δ-elemene (t_R 24.14 min).

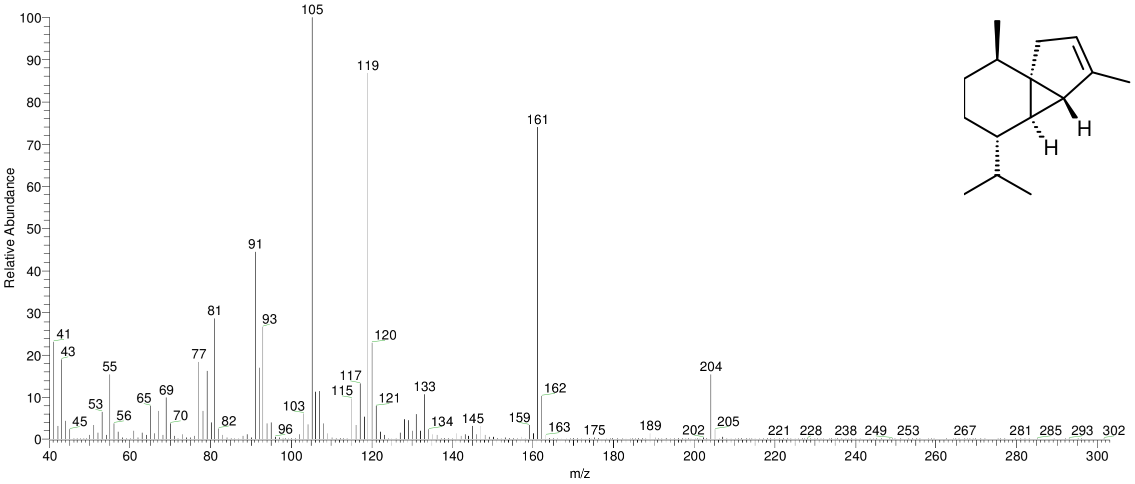

Figure S3. Mass spectrum of α-cubebene (t_R 24.56 min).

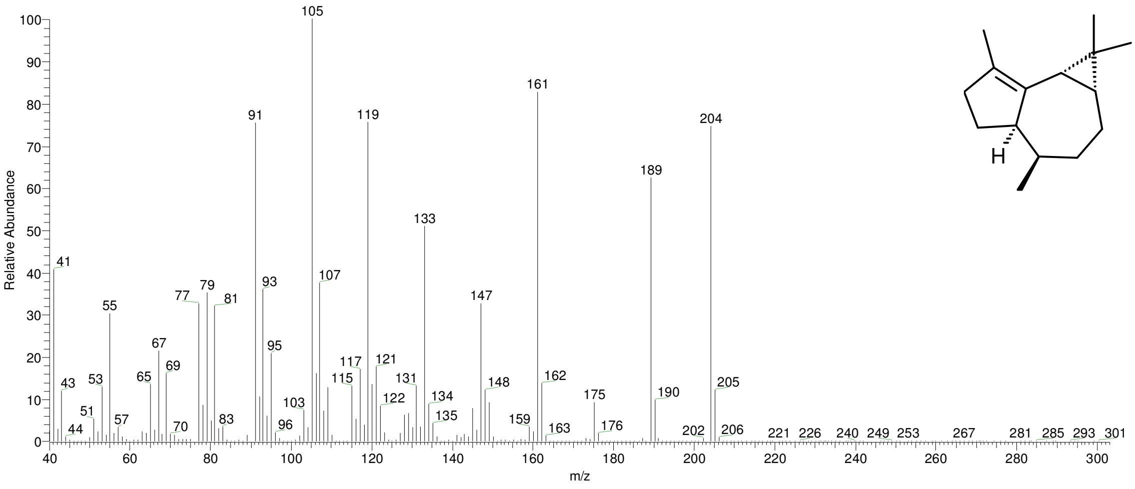

Figure S4. Mass spectrum of α-gurjunene (t_R 26.48 min).

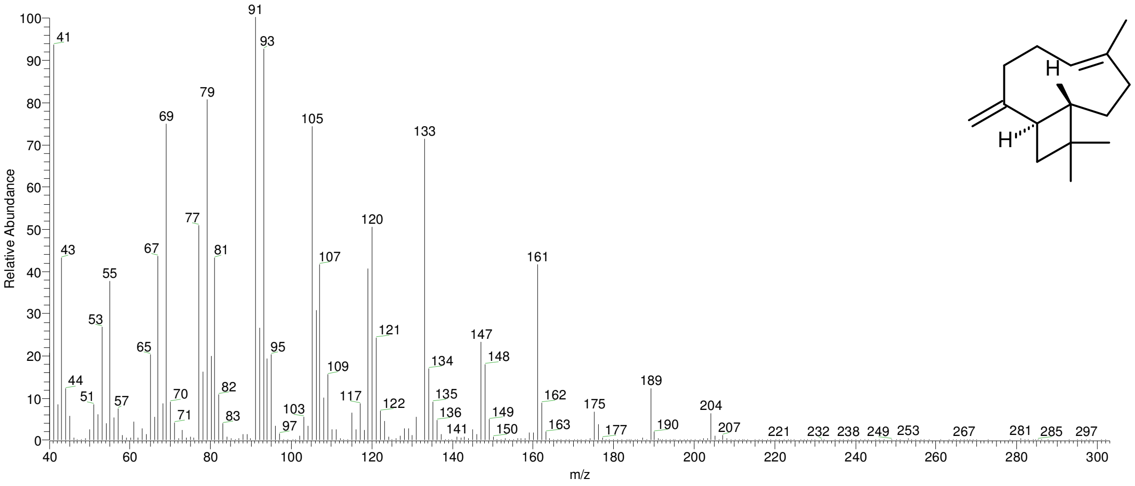

Figure S5. Mass spectrum of (*E*)-caryophyllene (t_R 26.76 min).

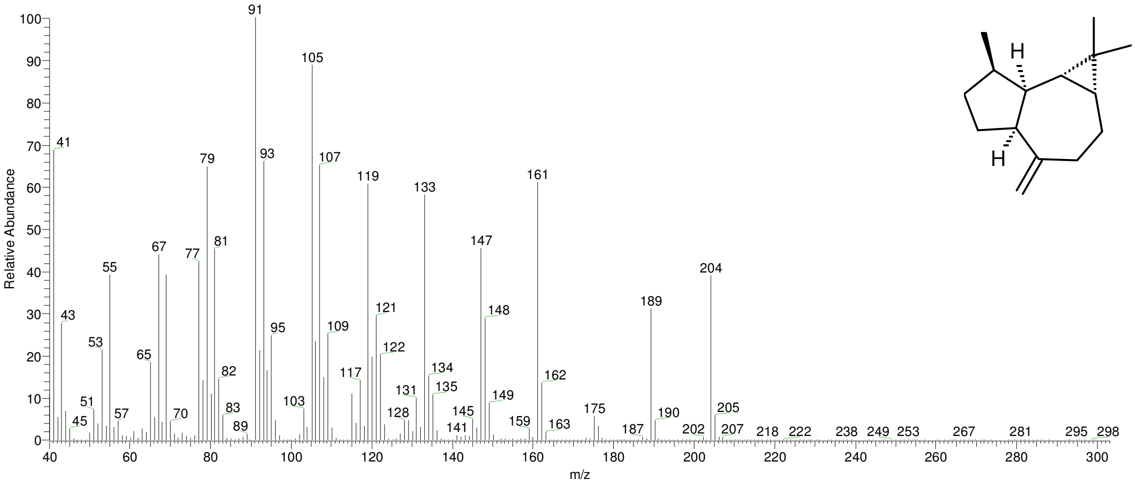

Figure S6. Mass spectrum of *allo*-aromadendrene (t_R 28.04 min).

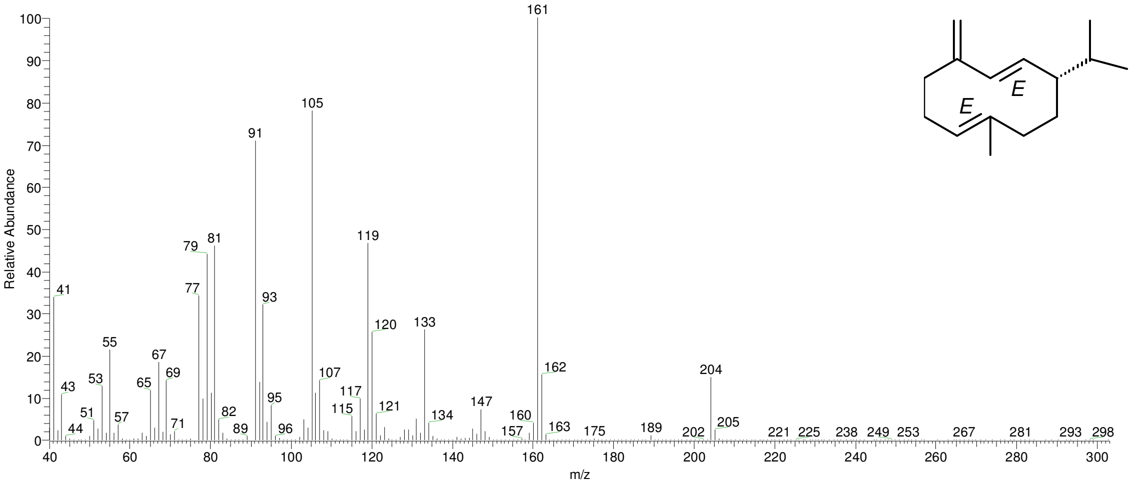

Figure S7. Mass spectrum of germacrene D (t_R 28.68 min).

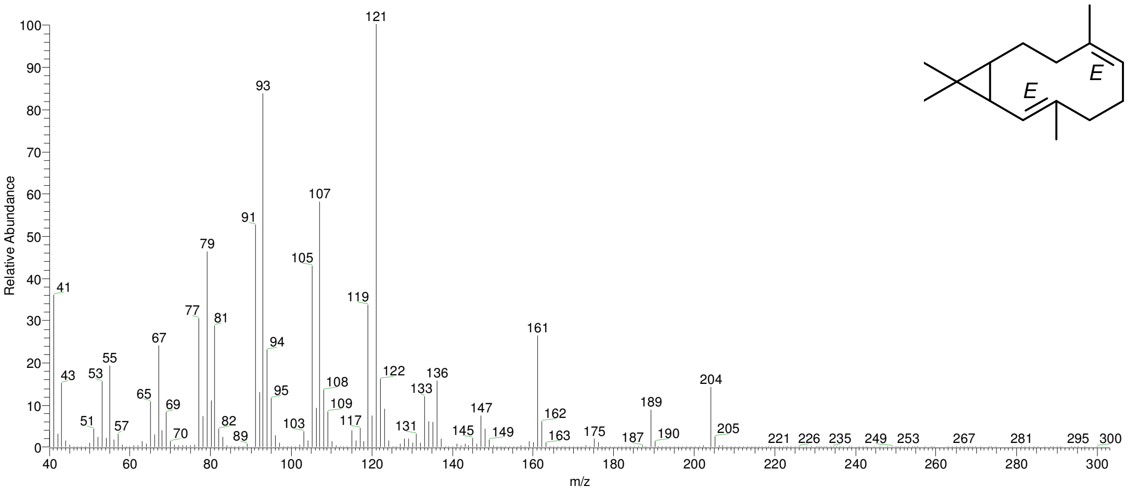

Figure S8. Mass spectrum of bicyclogermacrene (t_R 29.16 min).

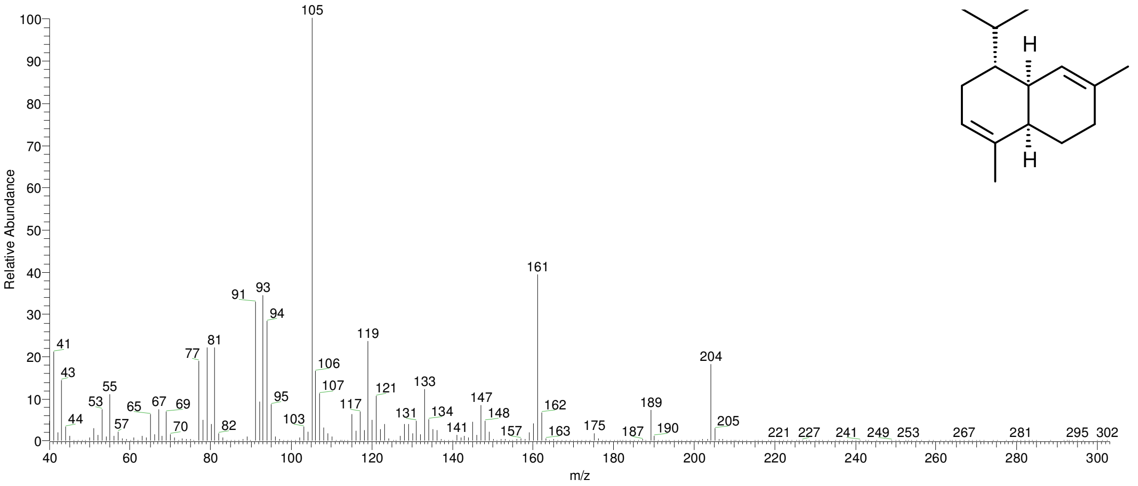

Figure S9. Mass spectrum of α-muurolene (t_R 29.28 min).

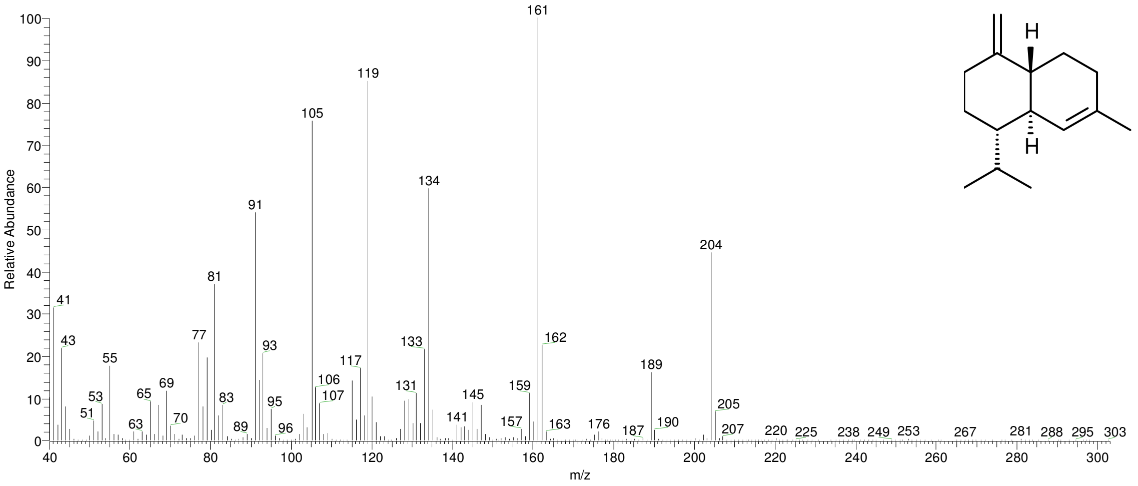

Figure S10. Mass spectrum of δ-cadinene (t_R 29.97 min).

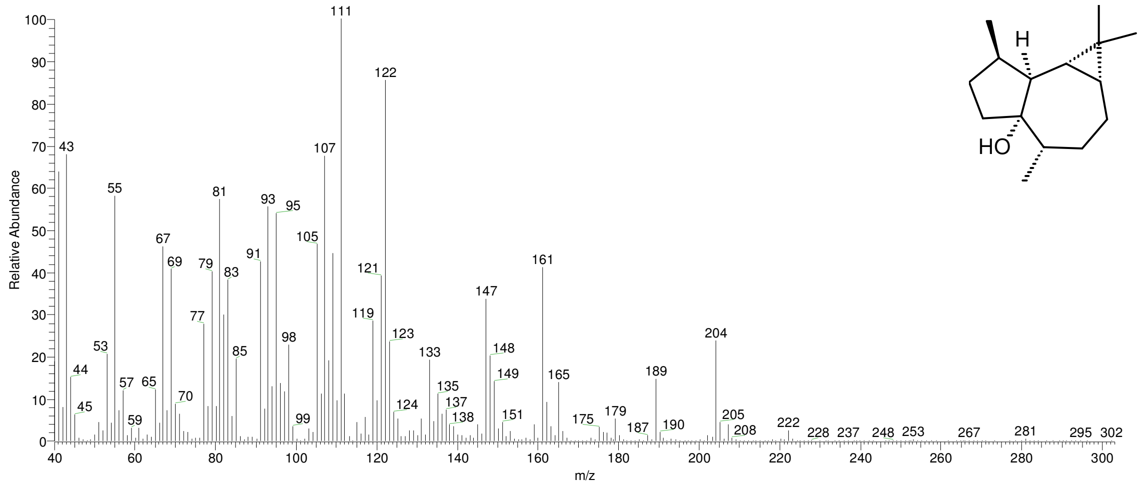

Figure S11. Mass spectrum of palustrol (t_R 31.23 min).

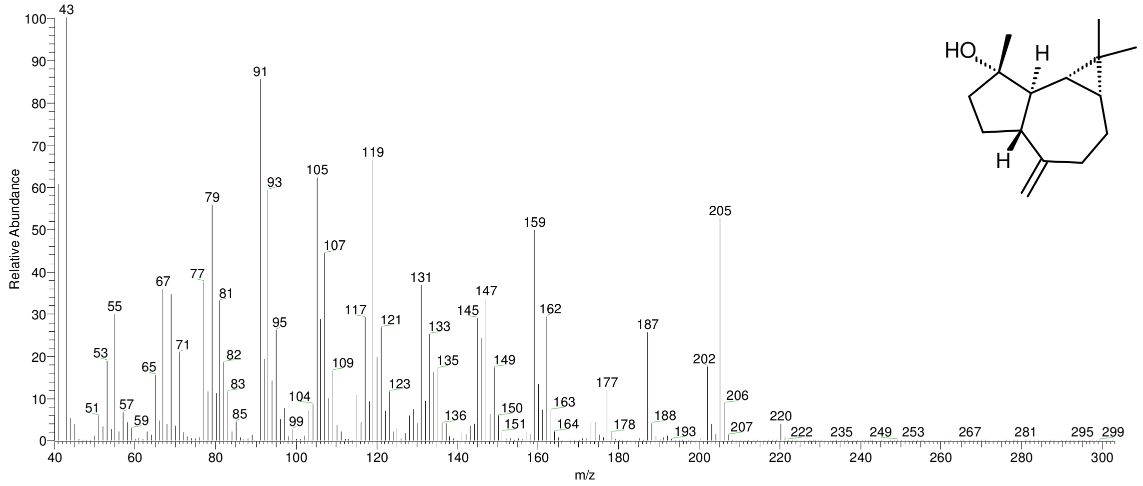

Figure S12. Mass spectrum of spathulenol (t_R 31.51 min).
